# Neoadjuvant Ifosfamide and Epirubicin in the Treatment of Malignant Peripheral Nerve Sheath Tumors

**DOI:** 10.1155/2017/3761292

**Published:** 2017-05-04

**Authors:** Angela C. Hirbe, Pippa F. Cosper, Sonika Dahiya, Brian A. Van Tine

**Affiliations:** ^1^Division of Medical Oncology, Department of Medicine, Washington University School of Medicine, 660 South Euclid Ave., St. Louis, MO 63110, USA; ^2^Siteman Cancer Center, St. Louis, MO 63110, USA; ^3^Department of Radiation Oncology, Washington University School of Medicine, St. Louis, MO 63110, USA; ^4^Department of Pathology and Immunology, Washington University School of Medicine, Campus Box 8118, 660 South Euclid Ave., St. Louis, MO 63110, USA

## Abstract

*Background and Objectives. *Malignant peripheral nerve sheath tumors (MPNSTs) are aggressive soft tissue sarcomas with poor overall survival. Response to chemotherapy has been debated for these tumors.* Methods.* We performed a retrospective analysis of the patients at our institution with a biopsy-proven diagnosis of MPNST that underwent neoadjuvant chemotherapy prior to surgery.* Results. *We retrospectively identified five patients who received neoadjuvant chemotherapy with epirubicin and ifosfamide that demonstrated a 30% reduction in tumor growth and a 60% response rate by RECIST criteria. Additionally, a metabolic response was observed in all three patients who received serial PET scans during neoadjuvant treatment. The clinical benefit rate, which includes stable disease, was 100%.* Conclusions. *Our data suggest that MPNSTs do respond to epirubicin and ifosfamide based chemotherapy and prospective studies are warranted to further define the clinical benefit.

## 1. Introduction

Malignant peripheral nerve sheath tumors (MPNSTs) are aggressive sarcomas, which account for approximately 5% of all soft tissue sarcomas [1]. Approximately 50% of MPNSTs occur sporadically or secondary to prior radiation therapy and approximately 50% arise in individuals with the Neurofibromatosis Type I (NF1) cancer predisposition syndrome [1–3]. In this regard, the prevalence of MPNSTs in the general population is approximately 0.001% compared to 0.1% in individuals with NF1. Composed of neoplastic Schwann cells, they most often arise from a benign precursor lesion (plexiform neurofibroma) in the setting of NF1 and the cumulative lifetime risk of these patients developing an MPNST is approximately 8–13% [3–5]. In the sporadic setting, the most well-known risk factor is previous radiation therapy [6–8]. For localized disease, the only known curative treatment involves surgery [9]. Radiation has been used to reduce the risk of local failure, although it does not affect overall survival [9]. Further, these cancers recur in ~50% of individuals and most die within five years, despite surgical resection.

Instituting effective therapies is one of the greatest challenges in managing MPNSTs. The use of chemotherapy to prevent recurrence has been vigorously debated without a clear answer. In addition, there is minimal published data available regarding the use of chemotherapy in the adjuvant and neoadjuvant setting for MPNSTs. The only prospective data demonstrated minimal responses to up-front chemotherapy with doxorubicin and ifosfamide, with response rates of approximately 17% (5/29 patients) in patients with NF1 and 33% (4/12) in the sporadic setting, leading to the notion that these tumors are minimally responsive to chemotherapy [10]. Similar response rates have been reported to chemotherapy in the metastatic setting for both NF1-associated and sporadic MPNSTs [11].

While the use of adjuvant radiation for soft tissue sarcomas to reduce the risk of local recurrence is a well-accepted treatment paradigm, the use of adjuvant or neoadjuvant chemotherapy in the treatment of soft tissue sarcomas, including MPNSTs, remains controversial [12–15]. The one study that showed a survival benefit to adjuvant chemotherapy in soft tissue sarcomas including MPNSTs utilized epirubicin as the anthracycline in the regimen [16]. Based on this study, our institutional practice is to give adjuvant or neoadjuvant epirubicin and ifosfamide for high-risk soft tissue sarcomas. Given the debate over the chemosensitivity of MPNST, we began to employ neoadjuvant chemotherapy when a tissue diagnosis is obtained prior to surgery, such that chemotherapy could be stopped early if the tumor was clearly refractory as assessed by continued growth during treatment. In the current study, we describe a series of five patients with MPNSTs that were treated in the neoadjuvant setting.

## 2. Materials and Methods

Approval for the collection of retrospective data regarding the treatment of sarcomas was approved by the Institutional Review Board at Washington University School of Medicine in St. Louis. The five consecutive patients with a biopsy-proven MPNST diagnosed prior to full surgical resection between 2012 and 2016 who were treated with neoadjuvant ifosfamide and epirubicin were selected for inclusion in this analysis. All patients received either a PET (*n* = 3) or CT scan (*n* = 2) at diagnosis, which was used to determine initial tumor size. Two patients received serial CT scans, two received serial PET scans, and one patient received both CT and PET to monitor response to neoadjuvant chemotherapy. The choice of which imaging test to utilize was dependent on insurance approval as not all insurance companies will approve PET scans for patients with sarcomas. PET scan would have been the preferred imaging modality based on our institutional experience. All tumor lesions were retrospectively “measurable” according to the RECIST1.1 definition [17]. Tumor size was determined by measuring the longest diameter in the axial or coronal plane. Tumor size was measured at the same anatomical location on each subsequent scan. A chart review was performed in order to obtain clinical and pathologic data for each patient. We were able to rereview the slides from the cases on Patients 2, 3, and 5 with a neuropathologist at our institution (Sonika Dahiya). Patients 1 and 4 were biopsied at an outside institution and those slides were not available for rereview. Percent treatment response was quantitated for those cases for which all slides were available for rereview.

## 3. Results 

**Figure 1:**
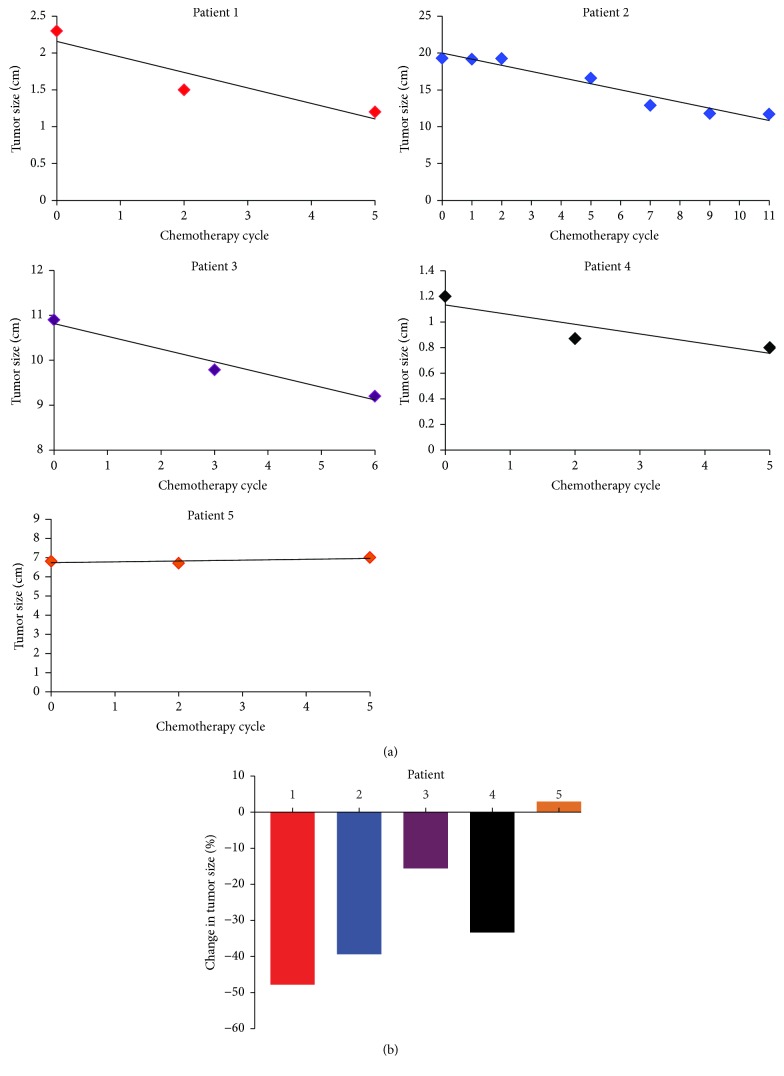
Effect of neoadjuvant chemotherapy on MPNST size. (a) Individual graphs are shown which depict the change in tumor size in five patients with MPNST treated with neoadjuvant epirubicin and ifosfamide. (b) Percent change in tumor size for each patient at completion of neoadjuvant chemotherapy.

**Figure 2:**
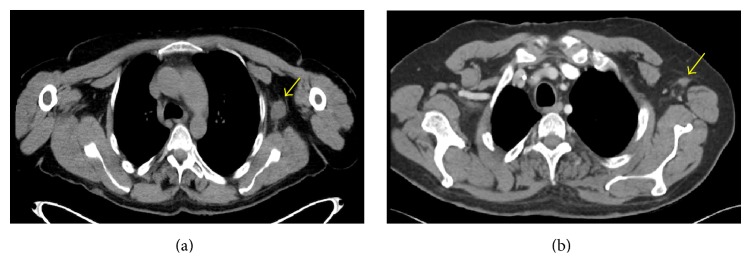
Treatment effect of an axillary MPNST following neoadjuvant chemotherapy. CT chest of Patient 1 revealing the left axillary MPNST (arrow) prior to treatment (a) and after five cycles of neoadjuvant chemotherapy (b). Note that patient's arms were not in the same position in each scan causing the tumor to be in a slightly different location.

**Figure 3:**
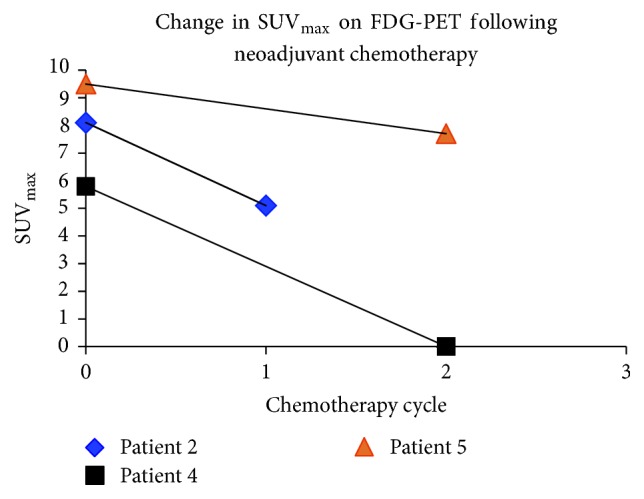
Change in SUV_max  _on FDG-PET. The change in SUV_max  _ of the MPNST on FDG-PET in three patients who received serial PET scans during treatment with neoadjuvant chemotherapy.

Five patients at our institution were treated with neoadjuvant chemotherapy consisting of 1800 mg/m^2^ ifosfamide on days 1–5 and 60 mg/m^2^ epirubicin on days 1 and 2 [16]. The characteristics of the patients are depicted in Table 1. Four males and one female were treated. The average age at diagnosis was 40 years. There were three patients with NF1 and two that were sporadic. Four patients are still alive at the time of this manuscript preparation and on average they are almost three years out from diagnosis. The absolute change in size of each individual's tumor is depicted in Figure 1(a). On average, we saw a 27% decrease in the size of the tumor following neoadjuvant chemotherapy (Figure 1(b)). This included 3 partial responses (PR), two from the sporadic MPNST patients and one from an NF1 patient, and 2 individuals with NF1 exhibiting stable disease (SD) by RECIST criteria, making the clinical benefit rate (CBR = PR + SD) 100%. Representative images of an axillary MPNST before and after neoadjuvant chemotherapy from Patient 1 reveal a 47% decrease in size (Figure 2). Additionally, for the patients from whom we were able to obtain serial PET scans, all three exhibited a metabolic response, including a complete metabolic response in Patient 4 (Figure 3).

From a clinical standpoint, these responses were dramatic. For example, Patient 3 was deemed unresectable at diagnosis. However, following neoadjuvant chemotherapy, the tumor was able to be removed with negative margins and on final pathology, extensive treatment effect was observed. Additionally, Patient 4 presented with significant pain and right upper extremity weakness. After two cycles of chemotherapy, the pain had improved dramatically and strength was returning to the arm. Following five cycles, the patient was back to baseline and there was no evidence of tumor by PET scan and the individual did not require surgery.

## 4. Discussion

In summary, we present several key findings of our retrospective analysis. First, we have shown a RR of 60% and a CBR of 100% in this small cohort of MPNSTs using neoadjuvant ifosfamide and epirubicin. This is higher than what is reported in most other studies in which the RR ranged from 17% to 45% depending on the study [9, 10, 18]. No studies to date have reported any difference in overall survival with chemotherapy in the treatment of MPNSTs [19]. However, most of these studies are small and retrospective in nature. Additionally, these reports pool data from multiple trials and multiple institutions. Interestingly, a recent study reported in abstract form saw a survival benefit in patients with high-risk soft tissue sarcoma including MPNSTs treated with neoadjuvant epirubicin and ifosfamide [20]. Future studies in our laboratory will employ genomic and proteomic analyses of tumors before and after chemotherapy in order to identify biomarkers that may predict response. Second, our data supports the notion that epirubicin may be the anthracycline that should specifically be used in the treatment of MPNSTs. However, future prospective studies would be necessary to test this hypothesis. While most regimens contain ifosfamide, the anthracycline used most often is doxorubicin. One of the dose-limiting toxicities of anthracyclines is the cardiac toxicity that can occur. There is both preclinical and clinical data that higher doses of epirubicin can be given with less risk of cardiac toxicity compared to doxorubicin. This may be part of the reason why a better effect is seen with epirubicin [21–23]. Finally, we see similar response rates using RECIST criteria in both sporadic and NF1-associated MPNSTs. This is in contrast to other studies which have demonstrated a far worse response rate for NF1-associated MPNSTs [18]. While previous studies have failed to show a definitive benefit to chemotherapy in the treatment of MPNST, our data would suggest that there is a role.

## 5. Conclusions

Taken together these data suggest that MPNSTs can be chemoresponsive tumors and that a well-designed adequately powered prospective trial utilizing epirubicin and ifosfamide is warranted to determine the true benefit to chemotherapy for this subtype of sarcoma. Following response rate in the neoadjuvant setting as well as overall survival may allow for a definitive answer in this regard.

## Figures and Tables

**Table 1:** Clinical features of patients.

Patient	Gender	Age at diagnosis	Tumor site	NF status	Initial path	Grade	Cycles of chemo	Decrease in tumor (%)	Final path	Clinical status	Overall survival (months)
Patient 1	M	59	Axilla	Yes	Neuroepithelial differentiation of MPNST	Unable to review	5	47	Residual fibroadipose tissue and metastatic sarcoma in 3/12 lymph nodes	NED	36

Patient 2	M	27	Mediastinum	Yes	MPNST	FNCLCC Grade 2	11	39	MPNST and areas of lung with pneumocyte hyperplasia	Deceased	14

Patient 3	M	29	RP	No	MPNST	FNCLCC Grade 3	6	15	MPNST with 80% treatment effect	NED	42

Patient 4	F	54	Brachial plexus	No	MPNST	FNCLCC Grade 2	5	33	CR without surgery	NED	43

Patient 5	M	35	Neck	Yes	MPNST	FNCLCC Grade 3	5	0	MPNST with 20% treatment effect	NED	9

RP = retroperitoneum.
